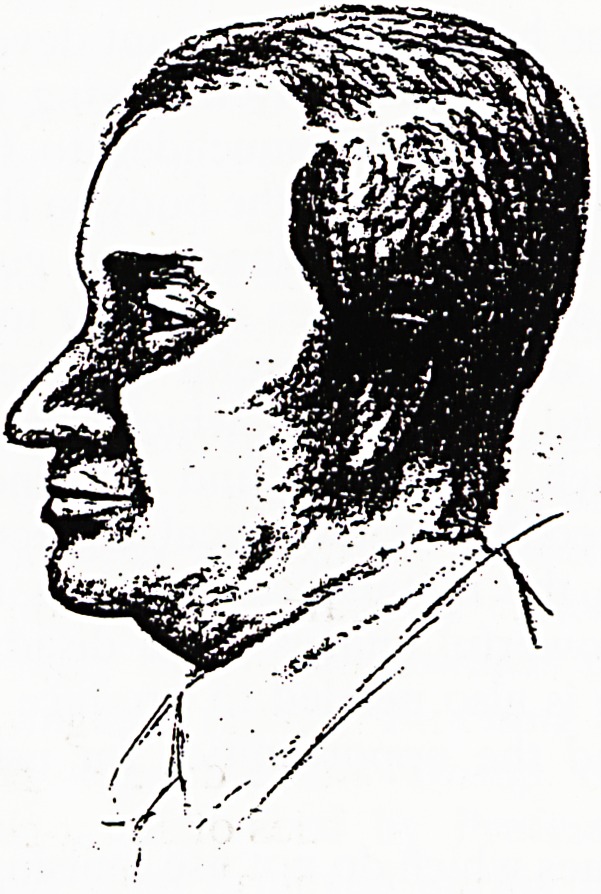# Bone Marrow Transplantation

**Published:** 1988-11

**Authors:** J. R. Hobbs

**Affiliations:** Department of Chemical Immunology, Charing Cross & Westminster Medical School, London


Bristol Medico-Chirurgical Journal Volume 103 (iv) November 1988

### Bone Marrow Transplantation can Correct Some Genetic Diseases and Leukaemias

**Professor J. R. Hobbs**
Department of Chemical Immunology, Charing Cross & Westminster Medical School, London

Professor Hobbs began by defending the right of the scientific and innovative doctors to do the good things that they think they can do in this day and age and let the politicians and others decide whether or not they will be adopted and financed. Workers should try to develop such advances to a cost effective level. The current climate in our universities and hospitals really has to be changed by fighting to stop the run down and he hoped that Bristol would be among the fighters.

The technique of getting marrow out from the interior of a bone through a needle was worked out in man in 1956 by the late Professor Humble at Westminster, before the Americans and before the French. Initially the sternum was used but it is a little thin and dangerous and he decided to go for the iliac crest and he worked out a technique by which a pint of bone marrow could be sucked out through four holes and given intravenously. This was the start of human marrow transplantation and in the following year this technique was used in Cambridge between identical twins, one of whom had aplasia, and they are both alive today, 30 years later! However, this can be questioned in the literature as the first transplant because as they are identical twins it is impossible to prove, according to the rigours of modern scientific analysis, that one sister has the marrow of the other. The French had their first survivors after the Yugoslav radiation accident in 1958, and the Americans theirs in 1959, but again unequivocal proof of permanent engraftment is lacking in their first successes.

The lessons then learnt were that you can't put another bone marrow in unless you suppress the natural immunology of the patient. Their white cells have to be poisoned with some kind of immunosuppression and they have to be in a state of no white cells for up to twenty days from the transplant before the graft takes.

During this time there is great risk of infection, a protective environment is necessary and supporting donations of white cells and platelets are used. Plastic bubbles were preferred at first but had to be given up because the nurses would not put up with them. Nowadays, the patient is in a room with a sterile airflow and everyone gowns-up to enter it. This avoids fungal infection which was one of the killers. The other big risk is that having denuded the patient of their immunology, you implant a new marrow which carries the immunology of the donor and this can start up 'graft versus host diseases' (GVH). Humble *et al.* did over 100 transplants from 1957 to 1966 with very few survivors because they knew nothing at all about tissue types, didn't know which donors they could use, and because even when they did autologous grafts, those were eventually followed by relapse of the malignant conditions treated. In 1968 the Americans, Good and Bach in Minneapolis, were the first to use tissue typing to choose a suitable donor and reduce the risk of GVH. Even with today's sophisticated tests, GVH cannot be entirely eliminated and so marrow transplantation can only be justified in diseases which would otherwise be fatal. To find a compatible sibling is the first search but their genes may carry the inherited disease and be unsuitable. In children today we can give a 95% guarantee of a successful transplant when a matched sibling donor can be found. When no such donor is available, the family is searched for a close match. Thereafter, unrelated volunteer donors can sometimes be found, but this is more expensive.

Results using donations from matched siblings in the treatment of genetic disease are very good. If the disease is corrected, and there is no death from infection in the first three years, long term survival can be expected; Good's patient is out 19 years since that first transplant, and our first — 17 years. They are both perfectly normal happy adults who have needed no medical attention since the year after their transplant. Matched sibling transplants are a joy and fully costed at today about £18,000 are extremely cost-effective. Using 1-genetic-haplotype-shared donors is a traumatic business; many patients are lost in the first year or two and the long-term success rate is about 40%, costing £45,000 for each survivor compared to an average of £120,000 in existing care before they would otherwise have died, so it is still cost-effective. Transplants for leukaemia are more expensive, about £25,000 a time using current methods, and not including the cost of inducing the remission. For acute myeloid leukaemia transplanted in first remission, European results from hundreds of patients show survivors relapse and die up to 7 years later, after which time about 45% seem cured, at a cost of about £56,000 per survivor. For second remission leukaemia, the results are below 25%, which means you have to graft at least 4 patients to get 1 survivor, costing about £100,000; in a real world I don't think my neighbours should have to contribute that sort of money so that I can live longer.

There are about 3,200 genetic diseases and something is known about the biochemistry of 1,500 of them. Of those there are about 100, or 7% of genetic disease, which might be treatable. The error has to be expressed in the bone marrow stem cells. The abnormal marrow of the patient has to be wiped out and totally replaced with a normal donor marrow, containing the normal expression for whatever was the error. The donor marrow takes over and will synthesise the normal component, to correct that error, (e.g. a missing enzyme) and at the same time will confer the immunology of the donor so that the recipient will now be tolerant to that product. There is the problem with gene transfer! In the test tube defective cells can be transfected to adopt to healthy genes and produce a gene product, but in a whole animal with an immunological system that gene product can, and often does, get neutralised. If we get a tolerant situation the bone marrow will produce a large number of leucocytes and we produce 50-300 gm of white cells a day, this is a very large amount of protein and a large amount of enzyme. With lysosomal enzymes these can often enter defective tissues, but getting into mitochondria (e.g. muscular dystrophy) may be very difficult or impossible. The patient will have had no experience of the missing enzyme and when this is introduced by the graft it can be antigenic. In 10% of cases antibody can be generated against the active site and in 90% it can bind to the enzyme and impair its delivery in the body. It is essential to prevent antibodies being formed; the recipient is shown what new protein is coming exactly 24 hrs. before we start the cyclophosphamide by giving the irradiated buffy coat of the donor. This can abrogate a primary immune response! In contrast, once the patient has been allowed to make antibodies it is almost impossible to eradicate a secondary immune response and the whole transplant is jeopardised.

The Westminster team have treated 100 children for otherwise fatal genetic diseases but time does not permit a fuller review, available elsewhere. Gaucher's disease is the big success story. The enzyme levels before transplant are negligible and afterwards they reach the levels of their donors and stay there for years after the graft. The Gaucher material in the bone marrow disappears within 5 months. The spleen has often been removed because it is so immuno-suppressive, but if left it reduces in size and so does the liver. Today, we elect to do a subtotal splenectomy before the transplant, leaving some tissue behind to enable primary immunological responses to encapsulated organisms (important under 5 years of age) to reduce post-splenectomy septicaemia. For Gaucher's disease there is a dramatic change in the child postgraft and they show wonderful catch-up growth.

Hurler's disease is another good example, a terrible condition in which the brain is damaged; the child deteriorates and becomes a vegetable but may not die until the age of 25. We have done 24 of these children now, and our longest survivors still look good. If they are transplanted under one year the lesions, mental retardation, blindness, deafness and so forth, will regress and the child lives a normal life, at any rate up to the age of 8, our oldest case. It is not a total cure however, as cartilage-formed bone still poses problems, especially where the donor was a heterozygote.

There are now 50 diseases1 which were formerly fatal with no alternative treatment that can now be corrected by this kind of transplant. There are 7 others, mostly mucopolysaccharidoses, where it is worth continuing to see what happens, and there are 3 outright failures — Niemann-Pick Type A, GM1 gangliosidosis and Pompé's disease.

The children should be done when they are in the best possible condition and not when they have deteriorated. With modern molecular biology you can often confirm the disease before it is too late and soon they will be classified better and related to the results of transplantation, which should be done before they have had multiple transfusions and become infected with cytomegalovirus, etc. Immunoprophylaxis must be practised unless it is certain no antibodies will be formed. A normal homozygote donor is preferred. Unrelated donor volunteers are often better than siblings who will often be carriers. This work must not be done without the necessary equipment and back-up facilities of a first-class paediatric set-up; today, it is probably unethical to do it in the side-ward of a general hospital.

**REFERENCES**

1. HOBBS, J. R. Displacement Bone Marrow Transplantation and Immunoprophylaxis for Genetic Diseases (1988) *Adv.Int.Med.* **33**, 81-118.

## Figures and Tables

**Figure f1:**